# A user-friendly machine learning approach for cardiac structures assessment

**DOI:** 10.3389/fcvm.2024.1426888

**Published:** 2024-07-05

**Authors:** Atilla Orhan, Hakan Akbayrak, Ömer Faruk Çiçek, İsmail Harmankaya, Hüsamettin Vatansev

**Affiliations:** ^1^ Department of Cardiovascular Surgery, Faculty of Medicine, Selcuk University, Konya, Türkiye; ^2^ Department of Pathology, Faculty of Medicine, Selcuk University, Konya, Türkiye; ^3^ Department of Biochemistry, Faculty of Medicine, Selcuk University, Konya, Türkiye

**Keywords:** anabolic-androgenic steroid, artificial intelligence, cardiac capillaries, image segmentation, machine learning, myocardial hypertrophy, myocardial hypertrophy in athletes

## Abstract

**Background:**

Machine learning is increasingly being used to diagnose and treat various diseases, including cardiovascular diseases. Automatic image analysis can expedite tissue analysis and save time. However, using machine learning is limited among researchers due to the requirement of technical expertise. By offering extensible features through plugins and scripts, machine-learning platforms make these techniques more accessible to researchers with limited programming knowledge. The misuse of anabolic-androgenic steroids is prevalent, particularly among athletes and bodybuilders, and there is strong evidence of their detrimental effects on ventricular myocardial capillaries and muscle cells. However, most studies rely on qualitative data, which can lead to bias and limited reliability. We present a user-friendly approach using machine learning algorithms to measure the effects of exercise and anabolic-androgenic steroids on cardiac ventricular capillaries and myocytes in an experimental animal model.

**Method:**

Male Wistar rats were divided into four groups (*n* = 28): control, exercise-only, anabolic-androgenic steroid-alone, and exercise with anabolic-androgenic steroid. Histopathological analysis of heart tissue was conducted, with images processed and analyzed using the Trainable Weka Segmentation plugin in Fiji software. Machine learning classifiers were trained to segment capillary and myocyte nuclei structures, enabling quantitative morphological measurements.

**Results:**

Exercise significantly increased capillary density compared to other groups. However, in the exercise + anabolic-androgenic steroid group, steroid use counteracted this effect. Anabolic-androgenic steroid alone did not significantly impact capillary density compared to the control group. Additionally, the exercise group had a significantly shorter intercapillary distance than all other groups. Again, using steroids in the exercise + anabolic-androgenic steroid group diminished this positive effect.

**Conclusion:**

Despite limited programming skills, researchers can use artificial intelligence techniques to investigate the adverse effects of anabolic steroids on the heart's vascular network and muscle cells. By employing accessible tools like machine learning algorithms and image processing software, histopathological images of capillary and myocyte structures in heart tissues can be analyzed.

## 1 Introduction

Artificial intelligence (AI) and machine learning (ML) techniques are increasingly used in medicine to improve the diagnosis and treatment of various diseases, including cardiovascular diseases (1). Automated techniques can make tissue analysis faster and more efficient, reducing the need for manual labor and saving time (2).

However, ML algorithms in medicine are new, and many researchers interested in using AI technologies may need more computer literacy or technical expertise. This is where image analysis platforms, such as ImageJ and Fiji, can be helpful (3, 4). These open-source software programs are easily extendable with plugins, scripts, pipelines, or workflows, allowing researchers with limited computing experience to develop and use ML algorithms.

Anabolic-androgenic steroids (AAS) have a wide range of clinically recognized uses. They are commonly used in heart transplant procedures to aid in the recovery process. AAS are also utilized in the treatment of osteoporosis to help increase bone density and reduce the risk of fractures. In addition, they are prescribed for individuals with chronic obstructive pulmonary disease to help improve muscle mass and strength. One of the most common indications for AAS therapy in men is hypogonadism, a condition where the body doesn't produce enough testosterone. AAS are also used in the treatment of catabolic states and cachexia, which are characterized by muscle wasting and weight loss. Furthermore, they may be prescribed as part of corticosteroid therapy to counteract the catabolic effects of long-term corticosteroid use. In addition to these uses, AAS have been employed in the treatment of depression, growth stimulation in male adolescence, prophylaxis of hereditary angioedema, and liver disease. They have also been used in the context of female-to-male transsexualism to promote the development of male secondary sexual characteristics. Furthermore, AAS have been investigated for their potential therapeutic effects in conditions such as multiple sclerosis, hypoplastic anemia, sexual dysfunction, and osteoporosis (5, 6). Anabolic-androgenic steroid (AAS) abuse is common among athletes and bodybuilders. There is strong evidence that co-administration of AAS with exercise can have negative effects on ventricular myocardial capillaries (7). However, most studies on this topic have relied on qualitative results and have a high margin of bias (2, 4). This highlights the importance of utilizing quantitative analyses to demonstrate the negative effects of AAS on the heart. AI and ML can be valuable tools in investigating these effects.

In this paper, we present a simple and user-friendly approach using ML algorithms to measure the effects of exercise and AAS on cardiac ventricular capillaries and myocytes in an animal model. This approach includes measuring the morphological features of capillary and myocyte structures from images obtained from the rats using the Trainable Weka Segmentation (TWS) plugin from Fiji (8). ML and TWS techniques, successfully utilized in analyzing various tissue structures, can also be effectively employed in the segmentation and morphological analysis of heart tissue structures such as capillaries and myocytes (9). Furthermore, we argue that even researchers without extensive programming and computer science knowledge can utilize artificial intelligence techniques to investigate the potentially detrimental effects of AAS on heart capillaries and myocytes in their studies.

## 2 Material and method

### 2.1 Ethical issues

This study followed “Experimental Animal Ethical Care” protocols approved by our University's Ethics Committee (27.04.2018-2018-15). The research center provided Male Wistar rats, kept in plastic cages at a temperature of 23 ± 2°C, 50% ± 10% relative humidity, and a 12-h day/night cycle while having *ad libitum* access to food and water. All rats used were male, and gender didn't affect the analysis.

### 2.2 Study design

The present study randomly divided Wistar-Albino male rats (*n* = 28) into four groups. Group I served as the control group and comprised sedentary animals without treatment with AAS. Group II consisted of animals that underwent an exercise protocol but did not receive AAS treatment. Group III included sedentary animals treated with AAS, while Group IV consisted of animals that underwent an exercise protocol and received AAS treatment. Following procedures, the animals were sacrificed by cervical dislocation under general anesthesia (Ketamine HCl 75 mg/kg and Xylazine 5 mg/kg).

### 2.3 Exercise procedure

For the exercise, an 8-lane treadmill specially designed for rats was used. At the beginning of the study, animals were placed in a 5-day acclimation period. Then, rats in exercise-related groups were given 45 min of exercise at 25 m/min 5 days a week for 6 weeks, as in the literature (10).

### 2.4 Anabolic androgenic steroid administration

We used Trenbolone enanthate as an AAS (Trenbolone E200, Pharma Generics). Trenbolone was administered intraperitoneally to groups III and IV rats, diluted in 100 ml peanut oil at a 10 mg/kg dose for different durations as in literature (10). The rats' body weights were measured at the start of the study and at the same time each week for the next 6 weeks, and dose adjustments were made for Trenbolone administration.

### 2.5 Histopathological procedure

After the animals had been euthanized, the rats' hearts were removed and fixed with a 10% formaldehyde solution, then embedded in paraffin blocks. Sections of 4 µm thickness were prepared from paraffin blocks with a microtome (Leica Microsystems EG 1130, Wetzlar, Germany). Samples were immunocytochemically stained with CD31 (monoclonal rat, JC70A; Dako, Glostrup; Denmark) using the automated Ventana BenchMark XT instrument. Finally, digital images were obtained at 100× magnification with a light microscope (Carl Zeiss Axio Imager D1, Carl Zeiss Microscopy, LLC, USA).

### 2.6 Image processing

**Figure 1:**
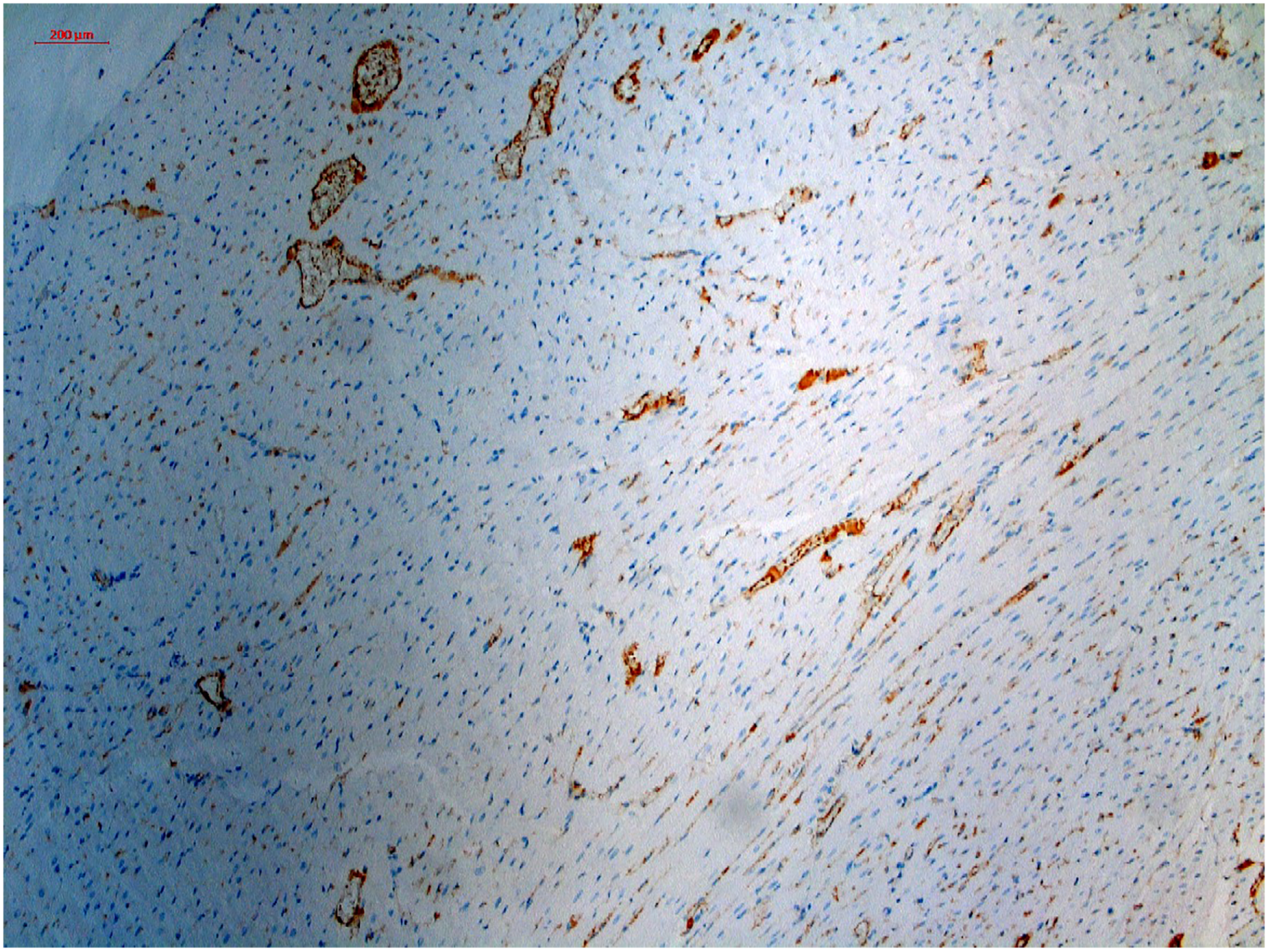
Histopathological specimen, immunohistochemically stained with CD-31 and digitalized and standardized at ×100 magnification with a light microscope.

All tissue sample images were resized to 1,167 × 874 pixels and standardized. A calibration scale was also inserted (Figure 1).

### 2.7 Machine learning and TWS training steps

This study developed an ML classifier using the TWS plugin v3.2.34 in ImageJ v2.1.0, included in the Fiji distribution. The TWS is an open-source toolkit that utilizes the Waikato Information Analysis Environment (WEKA) for ML and data mining. The default ML method in TWS is Fast Random Forest, which is implemented in the classifier. Two segmentation classes were defined: Capillary cells and Background, with all other artifacts and non-capillary cell images included in the Background class. The number of trees (numTrees) was set to 200 to optimize performance. The optimum settings were chosen based on the balance between image size, segmentation performance, and processing time.

The default features used were Gaussian blur, Hessian, Membrane projection, Sobel filter, Difference of Gaussians, membrane thickness = 1, membrane patch size = 19, minimum sigma = 1.0, maximum sigma = 16.0 and Fast Random Forest values: maxDepth = 0, numFeatures = 2, numTrees = 200. The classifier was trained using randomly selected image samples from each study group until the classification performance was maximized. In both the Fiji software and WEKA segmentation toolkits, the primary criterion for determining optimal settings and achieving near-perfect segmentation was the compatibility between the original image and the segmented image. The training process was considered complete only after obtaining approval from the consulted pathologist at every training stage. Training took four hours on a standard laptop computer (MacBook Pro 13-inch, 2017, Intel Core i5 @ 2.3 GHz, two cores, 8GB RAM).

After training, the classifiers and data files were saved and applied to all study samples. The segmented images were then refined with post-processing macro workflows and made suitable for morphological measurements (Table 1). Finally, morphological data were obtained from these refined images and saved in a data file for statistical processing.

The ML process was performed by creating two separate model files in the TWS. In the TWS plugin, the capillary structures were stained brown in the original image were labeled as Capillary, and everything else as Background (Figure 1). Classifier labels and sample images were trained by an experienced histopathologist and saved to the Capillary.model file and data to the Capillary_Data.arff file. Similarly, the myocyte nuclei stained dark blue in the original image (Figure 1) were labeled as the Nuclei, and everything else as the Background nuclei, and the model was recorded in the Nuclei.model file and the data in the Nuclei.arff file.

**Figure 2:**
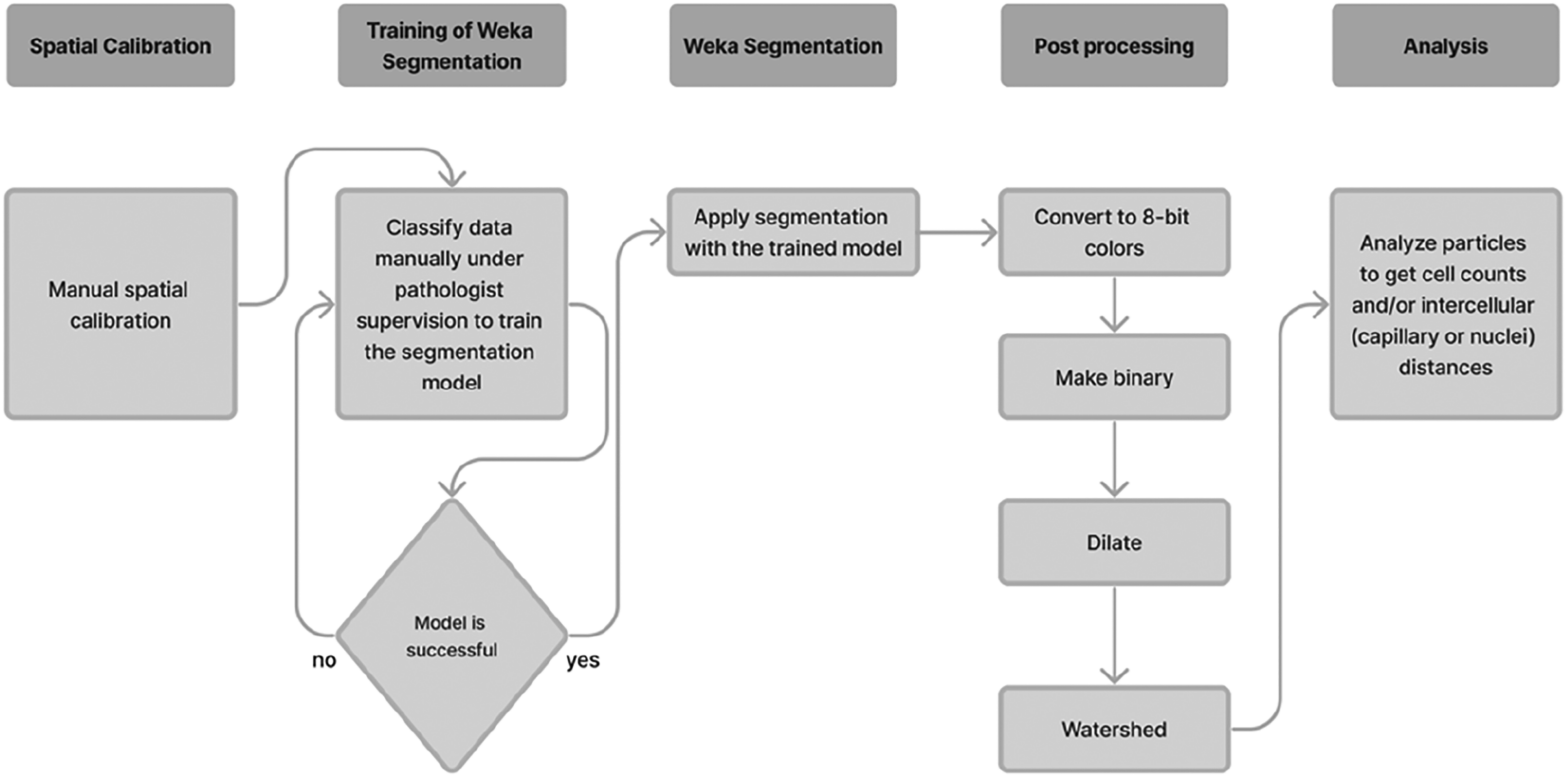
Detailed workflow for ML algorithm using trainable WEKA segmentation.

**Figure 3:**
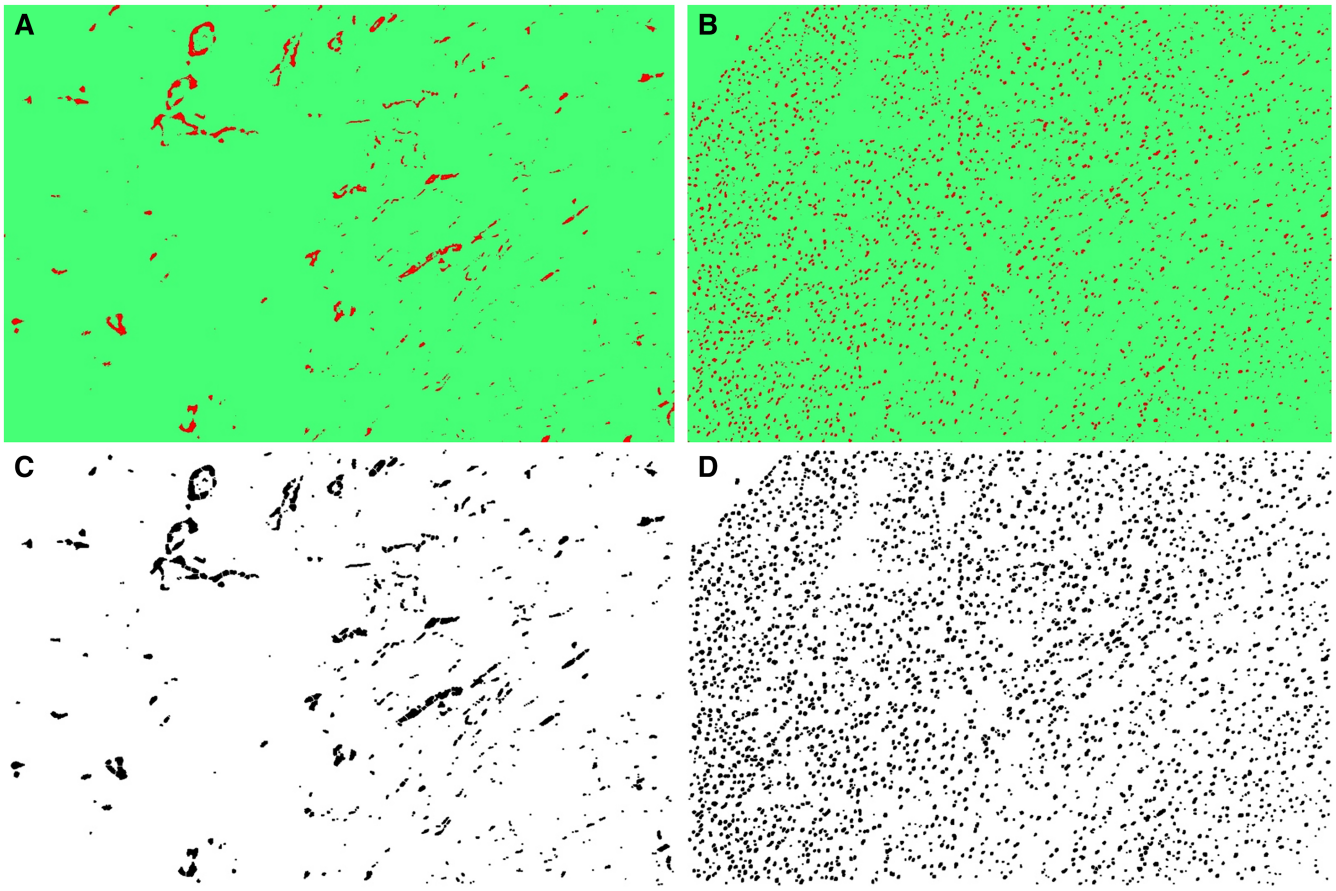
(**A**) The classified capillary images after the training of TWS, (**B**) the classified myocyte nuclei images after the exercise of TWS, (**C**) segmented capillary images obtained after running macro for post-processing, (**D**) segmented myocyte nuclei images obtained after running macro for post-processing.

Subsequently, the TWS plugin was employed to apply the trained model files to additional samples sequentially, leading to their segmentation according to the procedure outlined in Table 1, Figure 2. This yielded classified and segmented images of capillaries and myocytes, as demonstrated in Figure 3. Finally, post-processing macro steps were applied to the segmented images to obtain digital morphological data, which were saved in a data file.

**Table 1:** Step-by-step ML algorithm with trainable WEKA segmentation.

Spatial calibration
Open a study image with a calibration scale. Select the line selection tool to draw a straight line on the calibration scale. Go to menu analyze & set scale Enter the value on the calibration scale into the known distance field (The value in the study is 200 μm in all samples.) Set the units of the measurement (μm, mm or whatever) Check the “Global” option. Click OK. Finally, the spatial calibration settings are completed
Training a classification model
Select the workspace or whole space from the image Run the trainable Weka segmentation tool in Fiji (TWS plugin must be installed.) Rename classes (capillary and background) from settings. Select some capillaries with the freehand selection tool and add those traces to the capillary class. Select some background without capillary with freehand selection tool and add those traces to background class Click the “Train classifier” button and wait 2–3 min. After training, check if it works well. If it does not work well, add more traces and fix mistakes. Train again and redo previous steps if needed. Save classifier to a.model file Save data file to a.arff file //**The above steps must be repeated for myocyte nuclei.**
Applying classifier to another image
Open Image to apply RunTrainable Weka Segmentation Load Classifier >Select (your).model file Load Data File >Select (your).arff file Create Results
Post-Processing Macro Steps for the Classified Image
// **Selecting “Classified image” and 8 bit image conversion** selectWindow(”Classified image”); run(”8-bit”); //**Thresholding procedure** setThreshold(0, 120); setOption(”BlackBackground”, true); run(”Threshold…”); //**Binarization and segmentation** run(”Make Binary”); run(”Dilate”); run(”Watershed”); //**Morphological analysis of target class (Capillary, Myocyte or whatever)** run(”Analyze Particles…”, “ show=Masks display exclude summarize”); //**Calculation of intercapillary distances (Nearest Neighbor Distances Calculation (NND) plugin must be installed.** //**Click Analyze -> Set Measurements, Make sure “Centroid” is checked.** run(”Nnd “)

### 2.8 Statistical analysis

Data analysis was done with SPSS v22.0.0. A power analysis determined the sample size. Results show mean values and standard error of the mean. Statistical significance was *p* < 0.05. One-way ANOVA compared groups, with multiple group comparisons using Bonferroni *post hoc* test.

## 3 Results

### 3.1 Classification performance metrics

The automatic segmentation for capillary and myocyte nuclei detection showed high accuracy, with correctly classified instances for capillaries at 99.8223% and nuclei at 99.3464%. The performance of the TWS classifier was tested on both capillary and myocyte nuclei. A value of 1.0 in all performance measures indicates perfect classification. The precision value for the capillary model, the rate of over-prediction in capillary pixels, was 0.974. The recall value for the capillary model, the rate of under-prediction in capillary pixels, was 0.954. The accuracy of the TWS classifier for the capillary model, as measured by the F-score, was 0.964 (Table 2).

**Table 2:** Trainable WEKA^a^ segmentation performance summary for capillary and myocyte nuclei classes.

					Capillary			Myocyte nuclei	
Correctly classified instances					62,371 (99.8223%)			49,397 (99.3464%)	
Kappa statistic					0.9629			0.8924	
Mean absolute error					0.0059			0.0172	
Root mean squared error					0.0421			0.0736	
Relative absolute error					12.1332%			26.2749%	
Root relative squared error					27.0889%			40.6526%	
Total number of instances					62,482			49,722	
Confusion matrix for capillary and myocyte nuclei classes									
True class					True class				
Predicted class	Capillary		Background		Predicted class	Myocyte nuclei		Background_N	
	1,480 (TP^a^)		71 (FP^a^)			1,397 (TP)		291 (FP)	
	40 (FN^a^)		60,891 (TN^a^)			34 (FN)		48,000 (TN)	
Detailed accuracy by capillary and myocyte nuclei classes									
TP rate		FP rate	Precision	Recall	F-score	MCC	ROC area	PRC area	Class
0.954		0.001	0.974	0.954	0.964	0.963	1.000	0.992	Capillary
0.999		0.046	0.999	0.999	0.999	0.963	1.000	1.000	Background
0.998		0.045	0.998	0.998	0.998	0.963	1.000	1.000	Weighted avg.
0.828		0.001	0.976	0.828	0.896	0.896	0.998	0.977	Myocyte nuclei
0.999		0.172	0.994	0.999	0.997	0.896	0.998	1.000	Background nuclei
0.993		0.167	0.993	0.993	0.993	0.896	0.998	0.999	Weighted avg.

^a^ WEKA, Waikato information analysis environment; TP, true positive; TN, true negative; FP, false positive; FN, false negative.

Similarly, the precision value for the nucleus model, the rate of over-prediction in nuclei pixels, was 0.976. The recall value for the nucleus model, the rate of under-prediction in nuclei pixels, was 0.828. The accuracy of the TWS classifier for the nucleus model, as measured by the F-score, was 0.896 (Table 2).

**Figure 4:**
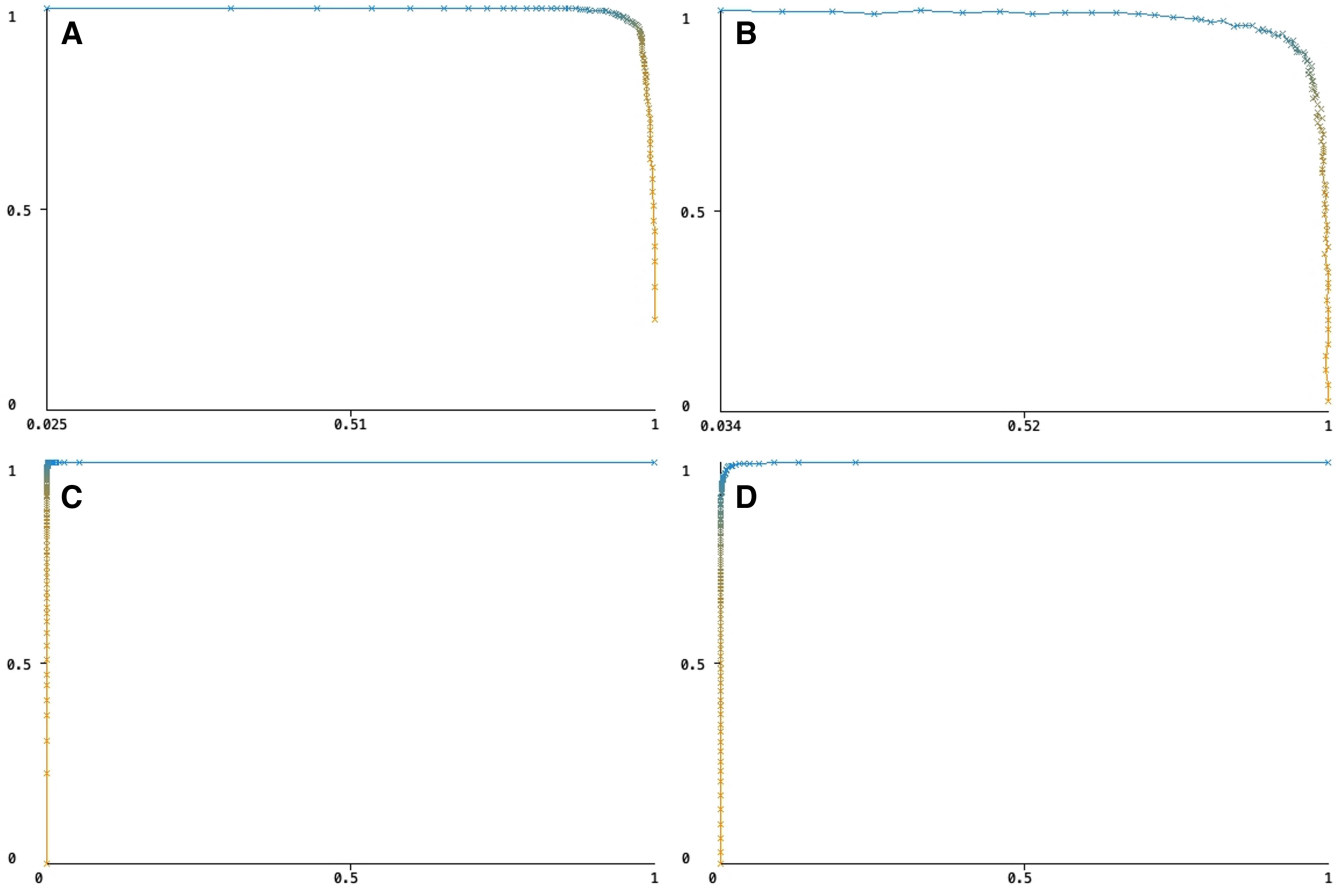
(**A**) The precision/recall trade-off for capillary pixels, (**B**) the precision/recall trade-off for myocyte nuclei pixels, (**C**) the ROC curve for capillary pixels, (**D**) the ROC curve for myocyte nuclei pixels.

The Precision/Recall trade-off was 1.02 for capillary pixels and 1.18 for nuclei pixels, and ROC curves were 0.999 for capillary pixels and 0.997 for nuclei pixels (Figure 4).

Overall, the performance of both models was close to perfect, with the capillary model outperforming the nucleus model.

### 3.2 Experimental results

The Results are shown in Table 3. The study investigated the effects of exercise, AAS administration, and their combination on heart tissue in rats. The ML was used to analyze heart tissue histopathological samples and measure capillary density, intercapillary distance (ICD), myocyte nuclei density, and internuclear distance (IND).

**Table 3:** Results of the study on the effects of different treatment groups.

	Group I (*n* = 7)	Group II (*n* = 7)	Group III (*n* = 7)	Group IV (*n* = 7)	*p*-value
	Mean ± SEM	Mean ± SEM	Mean ± SEM	Mean ± SEM	
Height rat body (cm)	39.4 ± 0.5	38.2 ± 0.7	38.2 ± 0.8	38.4 ± 0.5	0.54
Wh (g)	1.15 ± 0.07	1.12 ± 0.05	0.99 ± 0.04	0.96 ± 0.03	0.02*
Wb (g)	295.1 ± 12.7	273.4 ± 9.2	275.1 ± 21.4	268.3 ± 8.7	0.39
Wh/Wb	0.004 ± 0.005	0.004 ± 0.0001	0.004 ± 0.0001	0.004 ± 0.0001	0.05
Capillary count	478.7 ± 39.6	895 ± 89.9	717.9 ± 83.1	921.3 ± 97	0.005*
Myocyte nuclei count	3,655.3 ± 326.2	4,001.3 ± 187.1	3,396.6 ± 194.4	3,499.1 ± 222.7	0.14
Intercapillary distance	55.9 ± 1.9	44.9 ± 1.5	48.9 ± 2.7	45.3 ± 1.6	0.01*
Internuclear distance	30.1 ± 1	29.3 ± 0.46	30.9 ± 0.64	30.3 ± 0.69	0.31
Results that constitute a statistically significant difference between study groups					
	Group		Group		*p*-value
Capillary count	I		II		0.006*
			III		0.18
			IV		0.004*
	II		III		0.42
			IV		1
	III		IV		0.31
Intercapillary distance	I		II		0.004*
			III		0.09
			IV		0.005*
	II		III		0.49
			IV		1
	III		IV		0.59

The groups are designated as I (Control), II (Exercise), III (Anabolic-androgenic steroid), and IV (Exercise + Anabolic-androgenic steroid). Wh, represents the weight of the rat heart; Wb, represents the weight of the rat body and Wh/Wb, is the ratio between them.

* The *p*-value indicates the statistical significance of the differences between groups.

The study's results demonstrated the successful application of AI and ML in histopathological analysis. The ML algorithm could accurately segment and classify capillary and myocyte nuclei structures in the histopathological samples, enabling precise morphological measurements.

Accordingly, there was a significant difference between the groups in heart weight (*p* = 0.02). In addition, the data showed that exercise alone (Group II) significantly increased capillary density compared to the control (Group I) and the groups treated with AAS alone (Group III) or in combination with exercise (exercise + AAS) (Group IV) (*p* < 0.05). This indicates that exercise positively affects capillary density in heart tissue.

In contrast, the administration of AAS alone (Group III) did not significantly affect capillary density compared to the control group (Group I) (*p* > 0.05). However, in group IV, the administration of AAS attenuated the positive effect of exercise on capillary density. This suggests that AAS may hinder the beneficial effects of exercise on capillary density.

Furthermore, the ICD of the exercise group (Group II) was significantly shorter than in all other groups (*p* < 0.05). This indicates that exercise positively affects the spacing of capillaries in heart tissue. This trend was not observed in Group III and Group IV.

The data also showed no significant difference in the number of myocyte nuclei or IND among the groups (*p* > 0.05). This suggests that exercise, AAS administration, and their combination do not significantly affect these parameters in heart tissue.

All results showed that exercise benefits heart tissue, and AAS suppresses these effects. The study also demonstrated the potential of AI and ML for high accuracy in histopathological analysis.

## 4 Discussion

In this study, we used artificial intelligence and machine learning techniques to analyze the effects of the AAS and exercise on the heart in an animal model. Our results showed that exercise positively affected the heart in Group II compared to Group I. In contrast, the AAS (Group III) had negative effects, while AAS reduced the positive effects of exercise in Group IV. These findings align with previous research showing that AAS abuse can lead to structural and functional changes in the heart, including hypertrophy and fibrosis. On the other hand, exercise can improve cardiovascular health (6, 7).

The effects of the AAS and exercise on morphological parameters such as capillary and myocyte nuclei number and density, the ICD, and the IND in the myocardium at the histopathological level are still being determined by manual methods. These methods are prone to bias and do not provide a quantitative result. Additionally, analyzing large numbers of images is time-consuming and laborious for researchers (1, 11, 12).

Our research presents an automated approach for measuring the effects of medical treatments using ML algorithms. ML techniques are increasingly used in the medical field, particularly in the analysis and automation of histopathological images. Various studies have shown that they result in more objective outcomes (2, 4, 8, 9, 13–18).

This study proposes a simple and efficient approach for capillary segmentation using basic image processing techniques. The proposed method was evaluated on a dataset of rat capillary cell images, and it produced favorable results. Moreover, the technique exhibited robustness and dependability, even in challenging situations where manual segmentation was unfeasible or subject to subjective errors. Finally, quantitative assessment of the proposed method included F-score, precision/recall trade-off, and ROC curves, which were nearly optimal.

This study has the advantage that the proposed method requires minimal user intervention and can provide reliable results even under adverse viewing conditions. Therefore, the study suggests it is possible to measure capillary morphological measurements accurately and sensitively using ML and the proposed technique.

The AAS has various clinically recognized uses, including in heart transplant procedures, treatment of osteoporosis, chronic obstructive pulmonary disease, and in individuals infected with HIV. Hypogonadism is the most common indication for AAS therapy in men. The other clinical applications of AAS include the treatment of catabolic states and cachexia, corticosteroid therapy, depression, growth stimulation in male adolescence, prophylaxis of hereditary angioedema, liver disease, female-to-male transsexualism, multiple sclerosis, hypoplastic anemia, sexual dysfunction, and osteoporosis (5, 6). This treatment is sometimes combined with an exercise conditioning regimen (6, 7). Furthermore, these drugs are also used for experimental purposes in individuals who exercise (7). Additionally, some competitive athletes and individuals participating in various forms of physical exercise misuse AAS (7). However, the use of AAS can have negative impacts on cardiovascular health, such as disrupting lipid and lipoprotein metabolism (6, 7), increasing peripheral vascular resistance, and augmenting atherosclerosis, leading to abnormal blood, thrombosis, impaired coronary flow and perfusion, overstimulation of the sympathetic nerves of the heart, ultrastructural damage to heart muscle cells, direct toxic effects in heart muscle cell cultures and cardiovascular complications in athletes (6, 7). AAS use can cause cardiovascular complications, often due to illegal use (6).

During exercise, the heart needs more oxygen. Capillary beds help the heart get enough oxygen. AAS can disrupt capillarization in skeletal muscle during exercise. Not much is known about the effect of AAS on the heart's microvasculature (6). In various studies, intense exercise has not resulted in cardiac hypertrophy, despite an initial increase in heart weight observed during the first weeks of training (6). However, other studies have reported that the heart does exhibit hypertrophy in response to various forms of exercise (6). Some research has also suggested that AAS use can lead to hypertrophy (19). However, most studies on this topic need more in-depth qualitative analysis and numerical data. Additionally, the hypertrophic effects of AAS use on heart muscle cells and capillaries under training conditions have yet to be fully quantified.

Increased capillary number and density in the myocardium are well-established adaptations in response to physical exercise (6, 7). Studies have shown that increased capillary number and density increase the capillary-to-fiber ratio, ultimately decreasing oxygen diffusion distance (20). In our study, exercise positively affected the capillary number and density, while using AAS had a negative impact. In Group IV, AAS negated the positive effects of exercise on the capillary number and density.

The ICD and the IND are morphological measurements that provide valuable information about ventricular hypertrophy (21). To determine the ICD, we measured the distance between the centers of two neighboring capillary cells according to the Euclidean principle. This measurement was conducted using the Nearest-Neighbor Distances (NND) plug-in in Fiji (22), post-segmentation procedure, as part of a morphological analysis. Similarly, the IND was also calculated by determining the distance between the centers of two neighboring myocyte nuclei. These measurements increase due to the diameter of myocardial fibrils when there is ventricular hypertrophy (6, 7, 21). The ICD was significantly shorter in Group II compared to Group I (*p* = 0.01). This finding is consistent with previous studies. Like our findings, Tagarakis et al. (6, 7) argue that using AAS increases the ICD, which indicates ventricular hypertrophy. They found that the ICD did not cause a significant change in the AAS in the short-term study groups but did cause considerable prolongation in the long-term study groups.

We found the IND between groups to be insignificant, contrary to expectations. The limited sample size and short experimental time can explain this unexpected result at the IND. Also, studies show that regular exercise causes physiological changes in the ICD and the IND and does not cause pathological ventricular hypertrophy (6, 7).

One possible explanation for the harmful effects of AAS on the heart is that they alter the levels of hormones and growth factors in the body, leading to hypertrophy and fibrosis (23). On the other hand, exercise has been shown to improve cardiovascular health by increasing blood flow and promoting the growth of new blood vessels (24). However, our results suggest that combining AAS and exercise may harm the heart. This may be due to an imbalance in the hormones and growth factors that regulate cardiac function. These findings are consistent with previous studies, which have shown that co-administration of AAS and exercise can lead to increased oxidative stress and inflammation in the heart (19).

In our study, the heart and body weight of the rats were measured to evaluate the degree of hypertrophy. The ratio of heart weight to body weight (Wh/Wb) was calculated. Previous animal studies have suggested that the concomitant application of AAS and exercise training can increase heart weight (6, 25). Our statistical analysis revealed a significant difference in heart weight between the experimental and control groups (*p* = 0.02), with Group IV showing a higher heart weight. However, no significant difference was found in the Wh/Wb ratios between the groups. We attribute the lack of difference in the ratio to the limited sample size. Sample size limitation may not be sufficient to detect subtle changes in this ratio.

## 5 Limitations

A few limitations to our study should be considered when interpreting the results. First, we used a relatively small sample size, which may have limited our ability to detect significant differences between the groups due to the ethical committee decision. Second, our study was conducted in an animal model, which may not fully capture the complexity of human cardiovascular physiology. Indeed, this study is an animal study. We believe that further human-based research is necessary to evaluate the clinical applicability of its results. Finally, we only analyzed a few morphological features of the heart tissue, and there may be other changes that we did not measure that could be affected by AAS and exercise.

## 6 Conclusion

The study offers significant contributions to understanding the impact of AAS and exercise on cardiac health. It elucidates that exercise exerts beneficial effects on the heart, whereas the use of AAS and the concurrent administration of AAS and exercise can lead to unfavorable outcomes. These findings are noteworthy for athletes and bodybuilders contemplating using AAS to enhance performance. Furthermore, our study highlights the utility of ML algorithms in facilitating the automated analysis of heart tissue images, showcasing its potential for broader application to diverse diseases and tissue types. However, additional research is necessary to validate and expand our findings.

## Data Availability

The raw data supporting the conclusions of this article will be made available by the authors, without undue reservation.
